# The Effect of Stimulus Contrast and Spatial Position on Saccadic Eye Movement Parameters

**DOI:** 10.3390/vision7040068

**Published:** 2023-10-23

**Authors:** Viktorija Goliskina, Ilze Ceple, Evita Kassaliete, Evita Serpa, Renars Truksa, Aiga Svede, Linda Krauze, Sergejs Fomins, Gatis Ikaunieks, Gunta Krumina

**Affiliations:** 1 Department of Optometry and Vision Science, Faculty of Physics, Mathematics and Optometry, University of Latvia, LV-1586 Riga, Latvia; evita.kassaliete@lu.lv (E.K.); evita.serpa@lu.lv (E.S.); renars.truksa@lu.lv (R.T.); aiga.svede@lu.lv (A.S.); linda.krauze@lu.lv (L.K.); gatis.ikaunieks@lu.lv (G.I.); gunta.krumina@lu.lv (G.K.); 2 Institute of Solid State Physics, University of Latvia, LV-1063 Riga, Latvia; sergejs.fomins@lu.lv

**Keywords:** saccadic eye movements, saccadic eye movement parameters, stimulus parameters

## Abstract

(1) Background: Saccadic eye movements are rapid eye movements aimed to position the object image on the central retina, ensuring high-resolution data sampling across the visual field. Although saccadic eye movements are studied extensively, different experimental settings applied across different studies have left an open question of whether and how stimulus parameters can affect the saccadic performance. The current study aims to explore the effect of stimulus contrast and spatial position on saccadic eye movement latency, peak velocity and accuracy measurements. (2) Methods: Saccadic eye movement targets of different contrast levels were presented at four different spatial positions. The eye movements were recorded with a Tobii Pro Fusion video-oculograph (250 Hz). (3) Results: The results demonstrate a significant effect of stimulus spatial position on the latency and peak velocity measurements at a medium grey background, 30 cd/m^2^ (negative and positive stimulus polarity), light grey background, 90 cd/m^2^ (negative polarity), and black background, 3 cd/m^2^ (positive polarity). A significant effect of the stimulus spatial position was observed on the accuracy measurements when the saccadic eye movement stimuli were presented on a medium grey background (negative polarity) and on a black background. No significant effect of stimulus contrast was observed on the peak velocity measurements under all conditions. A significant stimulus contrast effect on latency and accuracy was observed only on a light grey background. (4) Conclusions: The best saccadic eye movement performance (lowest latency, highest peak velocity and accuracy measurements) can be observed when the saccades are oriented to the right and left from the central fixation point. Furthermore, when presenting the stimulus on a light grey background, a very low contrast stimuli should be considered carefully.

## 1. Introduction

Saccades are rapid eye movements that provide gaze shifting from one object of interest to another in both eyes simultaneously, thereby placing the object image on the central part of the retina—the fovea. Typically, saccades are initiated about 150 ms after the appearance of the visual stimulus and can last about 50 ms or less. Saccade duration depends on the characteristics of the stimulus and its parameters, as well as on the angular distance between the central fixation point and the stimulus presented in the visual periphery [1]. During this particular time interval, the gaze moves from one fixation point to another, allowing us to explore the external environment [2]. Saccades are characterized by latency, amplitude, peak velocity, accuracy, duration and other parameters. Experimental data indicate that the peak velocity of normal saccadic eye movements ranges from 200 to 600°/s [3], the average duration ranges from 30 to 120 ms [4], and the amplitude is usually below 15 degrees in normal viewing conditions [2]. The saccadic eye movement latency value can range from 100 ms to 1000 ms depending on the given task (voluntary, reflexive, anti-saccade task, etc.). The latency of reflexive saccades is on average 240 ms [5].

Several studies have demonstrated that saccadic eye movement performance can be affected by stimulus modality (e.g., visual, auditory and tactile targets), as well as different stimulus parameters—such as luminance [6], size [7], contrast [8], and stimulus direction [9]. When applying low-contrast visual stimuli, an increased latency of saccadic eye movements is observed, while saccadic eye movement accuracy is similar at both low and high contrast conditions [8,10]; downward saccades demonstrate higher latency measurements [11], and upward saccades tend to have a lower peak velocity [12].

Studies exploring the effect of age on saccadic eye movement performance have demonstrated that with age increased saccadic latency is observed when the target is presented to the left, up and down from the fixation target. The velocity of upwards directed prosaccades (saccadic eye movements in the direction of the stimulus) decreases with age and upward saccades become progressively more hypometric [13,14]. Also, the peak velocity of saccadic eye movements effectively reflects the level of arousal, making it a potentially valuable indicator in both ergonomics and clinical practice—saccadic peak velocity decreases with increased task difficulty [15].

Considering the wide application of saccadic eye movement analysis in the evaluation of the central nervous system performance, physiological and cognitive processes, as well as the increasingly extensive analysis of saccadic eye movements in other scientific fields, it is fundamentally important to create saccadic eye movement stimuli that would reduce the effect of stimulus parameters as much as possible. While all of the above mentioned studies have aimed to assess the effects of the same stimulus parameters (contrast and spatial position) on the same saccadic eye movement performance measurements (latency, peak velocity, acceleration (gain), average speed, amplitude, accuracy), all of these studies have applied different stimulus settings (color, shape, size, luminance, location) and background luminance. Furthermore, none of the abovementioned studies has explored the effect of stimulus parameters on the accuracy of saccadic eye movements, which is one of the main characteristics of saccadic eye movement performance.

Given the different stimulus parameters applied in studies analyzing saccadic eye movement performance at different contrast conditions and at different eccentricities, the aim of the current study is to perform a comprehensive study on saccadic movement performance (latency, peak velocity and accuracy) at different contrast conditions (including positive and negative contrast) and at different spatial positions. It is hypothesized that increased latency of saccadic eye movements will be observed under low contrast conditions, while the accuracy will not be significantly affected by different contrast conditions; downward-oriented saccades will have higher latency and upward-oriented saccades will have lower peak velocity measurements.

## 2. Materials and Methods

### 2.1. Participants

Fifteen participants (21 to 29 years old) were involved in the study. All participants had good visual acuity at near (60 cm) with or without contact lens correction, i.e., 0.8 decimal units. Participants who required spectacle correction were not included in the study. Participants did not report any neurological disorders that could affect saccadic eye movement performance. The study was approved by the Scientific Research Ethics Committee of the University of Latvia, and was carried out in accordance with the Declaration of Helsinki. All participants participated in the study voluntarily and were informed about the process of the current study, the eye tracking equipment, as well as their responsibilities and rights during the research process.

### 2.2. Equipment and Software

Gaze position on the computer screen and saccadic eye movements were detected with Tobii Pro Fusion (250 Hz) screen-based eye-tracking system operating in binocular mode. A Samsung S24C650PL 23.6 (1920 × 1080 px) 51.9 × 32.5 cm computer screen was used to present stimuli at a distance of 60 cm. Eye movement recording was performed by Tobii Pro Eye Tracker Manager and Tobii Pro Lab software, which are designed to conduct eye tracking experiments in Tobii system. Head position was fixed throughout the experiment using a chin rest.

### 2.3. Stimuli

The experiment started with a fixation cross (1 degree) located at the center of the computer screen. Right after the fixation cross disappeared, a 1 degree large circle was presented at one of four positions (up, down, left or right) relative to the position of the central fixation cross. The distance between the center of the fixation cross and the center of the saccadic eye movement stimulus was 10 degrees. Stimuli of negative and positive polarity were applied in the study.

**Figure 1:**
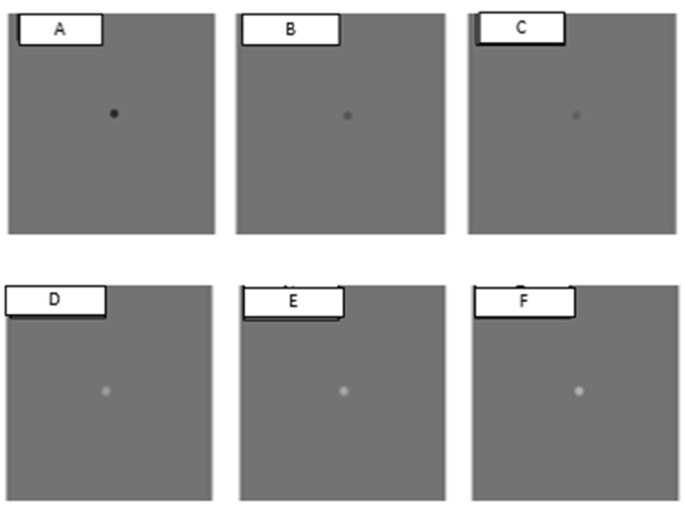
Saccadic eye movement targets presented on medium grey background (30 cd/m^2^). Three negative polarity contrast levels (Weber’s contrast values: (**A**) −0.83; (**B**) −0.67; (**C**) −0.33) and three positive polarity contrast levels (Weber’s contrast values: (**D**) 0.67; (**E**) 1.33; (**F**) 2.00).

**Figure 2:**
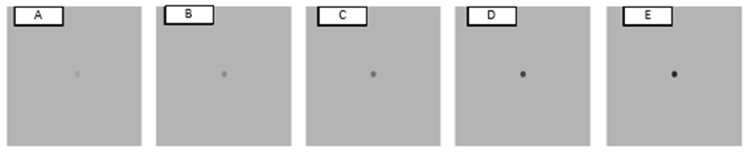
Saccadic eye movement targets on a light grey background (90 cd/m^2^). Five negative polarity contrast levels (Weber’s contrast values: (**A**) −0.22; (**B**) −0.44; (**C**) −0.67; (**D**) −0.89; (**E**) −0.94).

**Figure 3:**
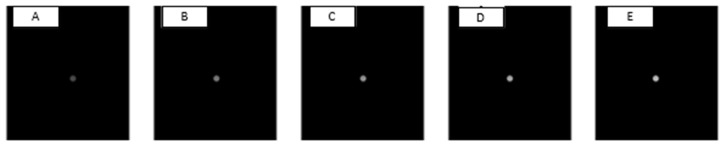
Saccadic eye movement targets on a black background (3 cd/m^2^). Five positive polarity contrast levels (Weber’s contrast values: (**A**) 2.33; (**B**) 9.00; (**C**) 15.67; (**D**) 22.33; (**E**) 29.00).

Saccadic eye movement targets were presented on either medium grey (positive and negative polarity), light grey (negative polarity) or black backgrounds (positive polarity). Medium grey background (30 cd/m^2^) with negative polarity had saccadic eye movement targets of 3 brightness levels of 5 cd/m^2^, 10 cd/m^2^ and 20 cd/m^2^ (the respective Weber’s contrast values −0.83, −0.67 and −0.33) and luminance of the fixation cross was 194.50 cd/m^2^; medium grey background with positive polarity had saccadic eye movement targets of 3 brightness levels of 50 cd/m^2^, 70 cd/m^2^ and 90 cd/m^2^ (the respective Weber’s contrast values 0.67, 1.33 and 2.00) and luminance of the fixation cross was 3.97 cd/m^2^ (Figure 1). For the light grey background (90 cd/m^2^), the luminance of the fixation cross was 194.50 cd/m^2^. The five luminance levels of saccadic eye movement targets against the light grey background were 70 cd/m^2^, 50 cd/m^2^, 30 cd/m^2^, 10 cd/m^2^, 5 cd/m^2^, with corresponding contrast levels of −0.22, −0.44, −0.67, −0.89 and −0.94 (Figure 2). For the black background stimuli (3 cd/m^2^), the luminance of the fixation cross was 3.97 cd/m^2^. Luminance levels for saccadic eye movement targets were 10 cd/m^2^, 30 cd/m^2^, 50 cd/m^2^, 70 cd/m^2^ and 90 cd/m^2^, with corresponding contrast levels: 2.33, 9.00, 15.67, 22.33 and 29.00 (Figure 3).

### 2.4. Procedure

Before the experimental part, all participants underwent the process of eye tracker calibration (5 points) and validation (4 points). Calibration and validation settings were similar to the experiment, i.e., matching the calibration background color (medium grey, black, or light grey) to the background color of the following experiment. After calibration and validation, participants were presented with saccadic eye movement targets of varying stimulus luminance and contrast levels in four different spatial positions in a mixed order. The presentation duration of each saccade stimulus was a constant 7 s, and the duration of the fixation stimulus was 3 s. The participants were instructed to change their gaze direction to the stimulus as quickly and precisely as possible and return to the fixation cross when the saccadic stimulus disappeared. Only monocular performance data of the dominant eye (Dolman test) were included in the following data analysis. After data acquisition, data on saccadic eye movement latency, accuracy, peak velocity in viewing all 64 stimuli were processed.

### 2.5. Data Analysis

Saccadic eye movement latency, average velocity and peak velocity were determined by Tobii Pro Lab software, applying Tobii Pro Lab one-parameter saccadic eye movement threshold identification (I-VT) filter which is based on the maximum velocity analysis [16]. The algorithm classifies eye movements into fixations and saccades based on the peak velocity threshold of 30°/s. If two consecutive gaze coordinates are at a minimum distance from each other (Tobii fix and ClearView fix filter) and the eye movement speed between the two records is below the maximum threshold level (30°/s), such data are considered to be a part of a one whole fixation. The opposite: if the movement speed is higher than the determined threshold value and the distance between two points is large, the corresponding data samples can be considered as a part of a saccade [17].

Saccadic eye movement data towards the stimulus were analyzed based on area of interest (AOI) data by Tobii Pro Lab software. AOI represented a pre-defined area on the fixation cross where the participants had directed their gaze right before the saccade execution. Saccade latency was determined based on AOI data on time to exit saccade—the saccade directed away from the area of interest (fixation cross) towards the saccadic eye movement target. Since the average latency of reflexive saccades is around 240 ms and saccades with latencies below 100 ms may have been anticipatory, only latencies between 100 ms and 300 ms were included in the further data analysis [5,18,19,20]. Peak velocity was determined based on the AOI data on peak velocity exit saccade representing the maximum velocity of the saccade directed from the fixation cross to the saccadic eye movement targets.

Saccadic eye movement amplitude and accuracy measurements were calculated based on the raw data on the gaze direction in time. The amplitude of saccadic eye movements was determined in Microsoft Excel as follows:

(1) $$\mathrm{Amplitude}=\sqrt{{\left(x_{2}-x_{1\;}\right)}^{2}+{\left(y_{2}-y_{1}\right)}^{2}}\;$$

where,

x_1_, x_2_—horizontal coordinates of eye fixation before and after the saccade (px);

y_1_, y_2_—vertical coordinates of eye fixation before and after the saccade (px).

The primary eye movement analysis data (raw data) were processed with Microsoft Excel and the accuracy of saccadic eye movements was determined as follows:

(2) $$\mathrm{Accuracy}=1-\frac{S_{e}}{T_{e}}$$

where,

$S_{e}$—landing error (cm);

$T_{e}$—distance between fixation position and target position (cm).

Landing error represents the magnitude of the vector between the coordinates of the gaze direction after saccade performance and the target location, meaning that the landing error depends on where the saccade target is placed (spatial position and distance). The landing error can be calculated by the Euclidean distance. It was calculated according to the formula:

(3) $$d_{Euclidian}\left(x,y\right)={\left(\sum_{i=1}^{k}x_{i}-y_{i}{}^{2}\right)}^{1/2}$$

where,

x_1_, x_2_—horizontal coordinates of eye fixation before and after the saccade (px);

y_1_, y_2_—vertical coordinates of eye fixation before and after the saccade (px).

In order to evaluate the effect of contrast on saccadic eye movement latency, accuracy and peak velocity, further statistical analysis was performed using the IBM SPSS 22 software. General Linear Model Repeated Measures multivariate analysis (SPSS Statistics) was applied to determine the effect of stimulus contrast level and direction on saccadic eye movement performance.

## 3. Results

### 3.1. Saccadic Eye Movement Latency as a Function of Stimulus Contrast Level and Spatial Position

**Figure 4:**
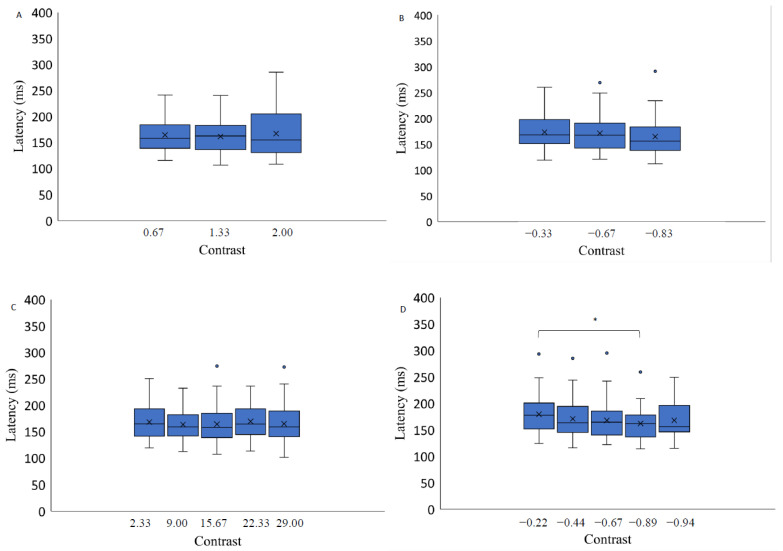
Saccadic eye movement latency as a function of stimulus contrast on three backgrounds (medium grey (positive (**A**) and negative (**B**) polarity), black (**C**) and light grey (**D**)). Data outliers, which are significantly different from the rest of the data, are marked with dots. * indicates statistically significantly different results after pairwise comparisons (post hoc analysis) with Bonferroni correction *p*-value < 0.05 (SPSS).

**Figure 5:**
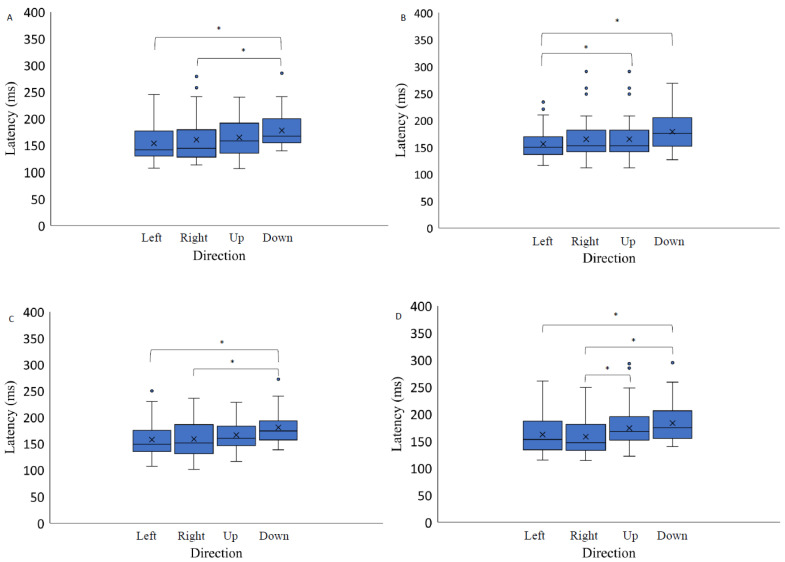
Saccadic eye movement latency as a function of stimulus direction on three backgrounds (medium grey (positive (**A**) and negative (**B**) polarity), black (**C**) and light grey (**D**)). Data outliers, which are significantly different from the rest of the data, are marked with dots. * indicates statistically significantly different results after pairwise comparisons (post hoc analysis) with Bonferroni correction *p*-value < 0.05 (SPSS).

Here we report the effect on stimulus contrast level and spatial position on different saccadic eye movement parameters. Since the number of different contrast levels varied between the three background conditions (light grey, medium grey, black), i.e., there were three or five different contrast levels, and the step of contrast values was not kept constant across the different backgrounds, further data analysis was performed for each background separately. Data were analyzed with a 3 × 2 repeated measures ANOVA with factors of target contrast (low, medium, high) and background color (grey, black). The results indicate a significant effect of the contrast level on saccadic eye movement latency only when the stimuli were presented on a light grey background (F(4,56) = 3.01, *p* = 0.03, η2 = 0.65): post hoc analysis (Bonferroni adjusted *p* value, *p*-value = 0.02) indicates higher latency measurements when the stimuli were presented at lower contrast levels (−0.22) compared to the highest contrast level (−0.89). The results of two-way repeated measures ANOVA (luminance level and spatial position factor) separately for each background revealed no significant contrast effect on the latency of saccadic eye movements when the target was presented on a medium grey background with positive polarity (F(1.44,20.21) = 0.62, *p* = 0.50, η2 = 0.06), on a medium grey background with negative polarity (F(2,28) = 2.13, *p* = 0.14, η2 = 0.12) and on a black background (F(2.80,39.24)) = 0.77, *p* = 0.51, η2 = 0.22) (Figure 4.). A significant effect of spatial position on the saccadic eye movement latency measurements was observed on all backgrounds: light grey (F(3,42) = 12.37, *p* < 0.05, η2 = 0.44); medium gray background with positive polarity (F(3,42) = 4.52, *p* = 0.01, η2 = 0.26); medium grey background with negative polarity (F(3,42) = 7.37, *p* < 0.05, η2 = 0.28); and black background (F(3,42) = 10.27, *p* < 0.05, η2 = 0.21) (Figure 5.).

Post hoc analysis indicated significant differences between the latency measurements when comparing saccadic eye movement performance to the right and up (Bonferroni adjusted *p* value, *p*-value < 0.05); to the right and down (Bonferroni post hoc: *p*-value < 0.05); and to the left and down (Bonferroni adjusted *p* value, *p*-value < 0.05) when the stimuli were presented on a light grey background. When attending the stimuli on a medium grey background with positive polarity, the cross-sectional data analysis indicated significant latency differences between two spatial positions—latency to the right was lower than when performing a saccade downwards (Bonferroni adjusted *p* value, *p*-value < 0.05) and latency to the left was also lower than when performing a saccade downwards (Bonferroni adjusted *p* value, *p*-value < 0.05). The analysis of saccadic eye movement performance when the targets were presented on a medium grey background with negative polarity revealed the following significant differences in latency measurements: when performing the saccade upwards and downwards the latency was higher than to the left: post hoc analysis (Bonferroni adjusted *p* value, *p*-value < 0.05). When attending the stimuli presented on the black background, cross-sectional data analysis indicated saccadic eye movement latency differences were lower when the saccade was performed downwards, comparing to the saccades to the right and to the left: post hoc analysis (Bonferroni adjusted *p* value, *p*-value < 0.05).

### 3.2. Accuracy of Saccades as a Function of Stimulus Contrast Level and Spatial Position

**Figure 6:**
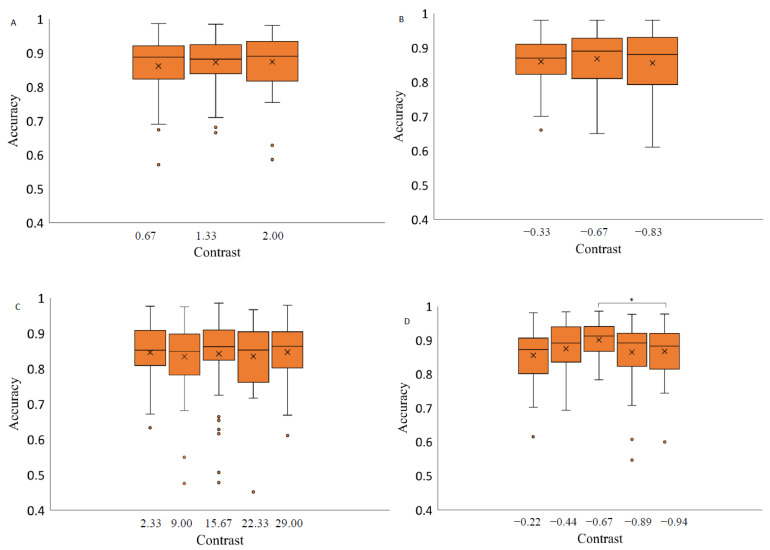
Saccadic accuracy as a function of stimulus contrast on three backgrounds (medium grey (positive (**A**) and negative (**B**) polarity), black (**C**) and light grey (**D**)). Data outliers, which are significantly different from the rest of the data, are marked with dots. * statistically indicates significantly different results after pairwise comparisons (post hoc analysis) with Bonferroni correction *p*-value < 0.05 (SPSS).

For each background, saccade accuracy was assessed separately depending on the contrast level of the stimulus and the spatial position of the saccadic eye movements. Data were analyzed with a 3 × 2 repeated measures ANOVA with factors of target contrast (low, medium, high) and background color (grey, black). The results indicate a significant effect of contrast level on saccadic eye movement accuracy when the stimuli were presented on a light grey background (F(4,56) = 4.66, *p*-value < 0.05, η2 = 0.35). Post hoc analysis revealed that the accuracy measurements at a medium contrast level (−0.67) were significantly higher than at the highest contrast level (−0.94) (Bonferroni post hoc: * *p*-value < 0.05). Two-way repeated measures ANOVA (luminance level and spatial position factor) separately for each background revealed no significant effect of contrast level on the saccadic eye movement accuracy when presenting the stimuli on a medium grey background with positive polarity (F(2,28) = 0.59, *p*-value = 0.56, η2 = 0.01), medium grey background with negative polarity (F(1.46,20.43) = 0.46, *p*-value = 0.58, η2 = 0.36) and on black background (F(4,56) = 0.55, *p*-value = 0.70, η2 = 0.25) (Figure 6).

**Figure 7:**
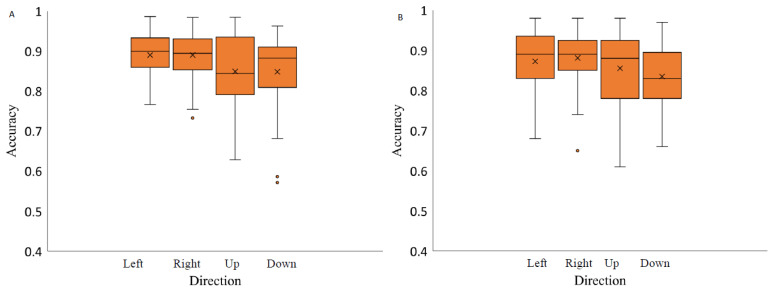

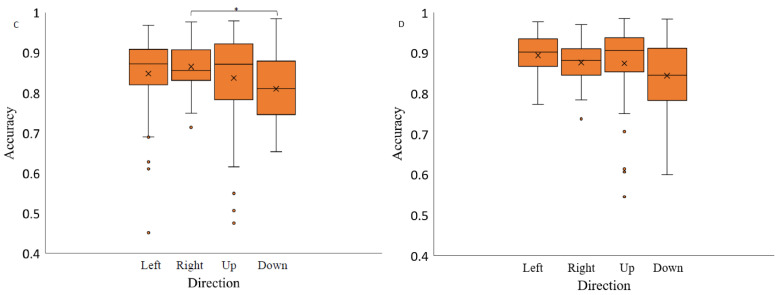
Saccadic accuracy as a function of stimulus spatial position on three backgrounds (medium grey (positive (**A**) and negative (**B**) polarity), black (**C**) and light grey (**D**)). Data outliers, which are significantly different from the rest of the data, are marked with dots. * statistically indicates significantly different results after pairwise comparisons (post hoc analysis) with Bonferroni correction *p*-value < 0.05 (SPSS).

The analysis of saccadic eye movement direction effect on the saccade accuracy revealed no significant effect when the stimuli were presented on light grey background (F(1.64,23.02) = 2.80), *p*-value = 0.09, η2 = 0.34), and medium grey background with positive polarity (F(1.66,23.23) = 2.69, *p*-value = 0.10, η2 = 0.10). Two-way repeated measures ANOVA analysis indicated on a significant effect of spatial position on saccadic accuracy when the stimuli were presented on medium grey background with negative polarity (F(3,42) = 3.04, *p*-value = 0.04, η2 = 0.22) and a black background (F(3,42) = 3.06, *p*-value = 0.04, η2 = 0.27) (Figure 7). Post hoc analysis revealed significant differences in accuracy measurements only between the saccades to the right and downwards and only when the stimuli were presented on black background (Bonferroni adjusted *p* value, *p*-value < 0.05).

### 3.3. Peak Velocity of Saccades as a Function of Stimulus Contrast Level and Spatial Position

**Figure 8:**
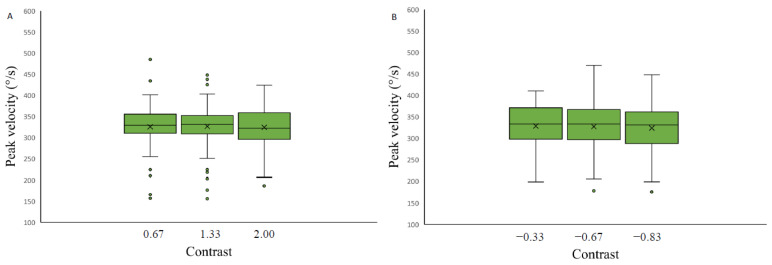

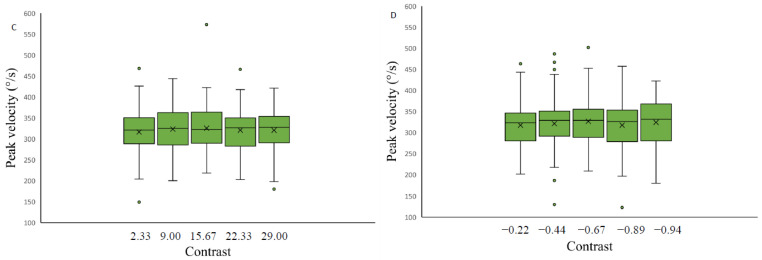
Saccadic peak velocity as a function of stimulus contrast on three backgrounds (medium grey (positive (**A**) and negative (**B**) polarity), black (**C**) and light grey (**D**)). Data outliers, which are significantly different from the rest of the data, are marked with dots.

The effect of stimulus contrast level and saccade spatial position on the saccadic eye movement peak velocity measurements was assessed separately for each of the four backgrounds. Data were analyzed with a 3 × 2 repeated measures ANOVA with factors of target contrast (low, medium, high) and background color (grey, black). The results revealed no significant contrast effect on saccadic peak velocity measurements on any of the four backgrounds applied: light grey (F(2.26,31.70) = 1.09, *p*-value = 0.35, η2 = 0.19), medium grey with positive polarity (F(2,28) = 0.07, *p*-value = 0.93, η2 = 0.12), medium grey with negative polarity (F(2,28) = 0.80, *p*-value = 0.46, η2 = 0.10) and on the black background (F (2.30,32.23) = 0.76, *p*-value = 0.49, η2 = 0.22) (Figure 8).

**Figure 9:**
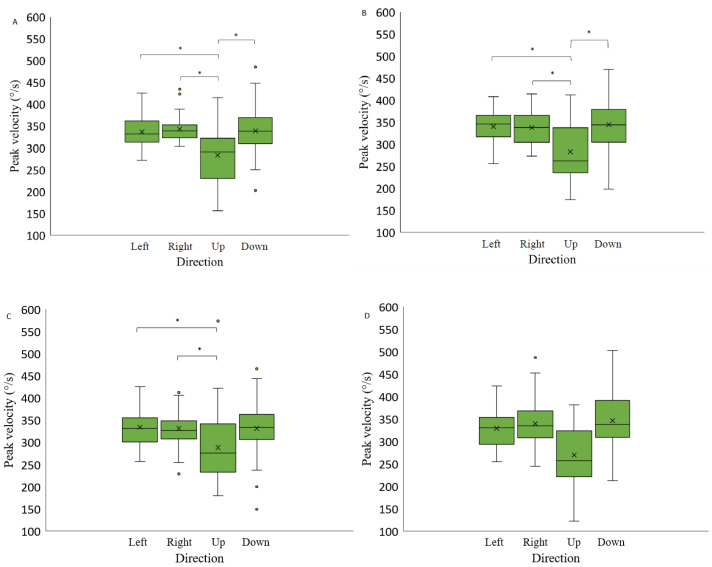
Saccadic peak velocity as a function of stimulus spatial position on three backgrounds (medium grey (positive (**A**) and negative (**B**) polarity), black (**C**) and light grey (**D**)). Data outliers, which are significantly different from the rest of the data, are marked with dots. * indicates statistically significantly different results after pairwise comparisons (post hoc analysis) with Bonferroni correction *p*-value < 0.05 (SPSS).

On the contrary, two-way repeated measures ANOVA (luminance level and spatial position factor) separately for each background a significant effect of spatial position on the saccadic peak velocity was observed when the stimuli were presented on a light grey background (F(2.22,31.10) = 24.47, *p*-value < 0.05, η2 = 0.17), on a medium grey background with positive polarity (F(1.73,3.73) = 10.34, *p*-value < 0.05, η2 = 0.45), on a medium grey background with negative polarity (F(1.71,23.87) = 8.89, *p*-value < 0.05, η2 = 0.46), and a black background (F(3,42) = 7.29, *p*-value < 0.05, η2 = 0.17) (Figure 9).

Post hoc analysis revealed that when the saccadic eye movement targets were presented on a medium grey background with a positive or negative polarity, the saccades performed upwards had higher peak velocity compared to other spatial positions (to the right, left, downwards) (Bonferroni adjusted *p*-value value, *p*-value < 0.05). When the stimuli were presented on the black background, the peak velocity of the upwards-directed saccades was slower when compared to horizontal saccades (to the right and to the left) (Bonferroni adjusted *p* value, *p*-value < 0.05).

## 4. Discussion

The current study explores the effect of stimulus contrast level and spatial position on saccadic eye movement performance. The obtained results indicate that a significant stimulus contrast effect on saccadic eye movement latency measurements can only be observed when the object is presented on light grey background. Furthermore, significant differences can be observed only when comparing the performance between the lowest contrast level and when the stimuli are presented on one of the highest contrast levels (Weber’s contrast value of −0.89). The results also demonstrated a significant effect of stimulus spatial position on saccadic eye movement latency measures at all background luminance levels included in the study (black, light gray, gray (with negative and positive polarity))—horizontal saccades had lower latencies regardless of the background color.

Previous studies have demonstrated that with increasing luminance or contrast levels saccadic eye movement latency tends to decrease [6,8]. The following parameters were applied in the study by Matsumiya and colleagues (2016) exploring the effect of stimulus contrast on saccadic latency and targeting error: stimulus brightness levels of 24.0 cd/m^2^, 25.1 cd/m^2^, 27.4 cd/m^2^, 32.0 cd/m^2^ and 41.1 cd/m^2^, and background brightness 22.8 cd/m^2^. By calculating the corresponding Weber’s contrast levels, it is possible to determine that previously mentioned study applied lower contrast stimuli (0.05, 0.10, 0.20, 0.40 and 0.80) than the current study [8]. On the contrary and similar to other studies evaluating the effect of contrast level on saccadic eye movement latency [21], the saccadic eye movement latency obtained in the current study did not change when stimuli were presented on a medium grey background with positive polarity and contrast levels of 0.67, 1.33, and 2.00. Also, in the case of other backgrounds (black background, medium grey background with negative polarity and light grey background), no tendency was observed for saccadic eye movement latency to decrease with increasing contrast. Significant differences in latency were only observed at the lowest contrast levels when displaying the object on a light grey background (90 cd/m^2^). Therefore, it is possible to conclude that at a sufficiently high contrast level, the contrast value no longer significantly affects the reaction time of the saccadic eye movements.

Dresp-Langley and Reeves (2014) demonstrated that color brightness has a significant impact on how we perceive objects and their backgrounds. This impact is observed in both relative distance and depth perception [22]. However, previous research exploring the relation between the accuracy of saccadic eye movements and target contrast levels on two-dimensional tasks [8] have not observed a significant target contrast effect on the saccadic eye movement accuracy. Since the current study has applied different levels of background luminance (light grey, medium grey with positive and negative polarity, as well as black background), the results expand the already available scientific information on the influence of various parameters on the characteristics of saccadic eye movements. The results obtained in this study demonstrate a significant stimulus contract effect on saccadic eye movement accuracy only when the stimuli are presented on the light grey background (90 cd/m^2^) and the contrast levels are −0.22, −0.44, −0.67, −0.89 and −0.94. On the contrary, no significant effect of the stimulus contrast on the peak velocity of saccades is observed at any of the background conditions.

Saccadic eye movement control is an interaction between the top-down (voluntary) and bottom-up (reflexive) processes of visual attention. In natural conditions, the attention is directed to the most salient regions of the visual field. The saliency map or the levels of salience of the visual information is considered to be based on image processing—the analysis of brightness, texture, borders and other visual parameters [23,24,25,26]. Clark (1999) presented a model of saccadic eye movement control, based on the target eccentricity and stimulus saliency, demonstrating that saccadic eye movement latency is lower for objects with higher saliency. While in the current study, the minimal contrast value of stimulus detection was not applied, the results demonstrate that brightness as an individual parameter does not cause significant differences in latency measurements. We can argue that higher latency measurements observed in low contrast conditions in other studies [8] might be related to the physiological processes of target detection in the peripheral visual field. However, once the stimulus is distinguishable, the latency results are not affected by the brightness or the saliency of the stimuli. A future study on the saccadic eye movement parameters with several simultaneously demonstrated stimuli of different brightness and contrast levels would give a more comprehensive insight into the latency control based on the stimulus salience [27].

The current study has also explored saccadic eye movement performance at different spatial positions (up, down, left and right). The obtained results demonstrate a significant effect of stimulus spatial position on saccadic eye movement latency and peak velocity measurements at all background luminance levels applied in the study (black, light grey, medium grey (with negative and positive polarity)). When presenting an object on a medium grey background with negative polarity and on a black background, saccade accuracy is also affected by saccade direction.

A significant spatial position effect on the peak velocity measurements was observed when the stimuli were presented on light grey background (90 cd/m^2^), on black background (3 cd/m^2^) and on medium grey background with both positive polarity and negative polarity (30 cd/m^2^). Similar to the results obtained by [10], the current study indicates that upward saccades are characterized by a lower peak velocity measurements than saccades in other directions, which could be explained by the eye movement limitations caused by the eyelids [28,29]. On the other hand, unlike the results obtained by [12] the current study does not demonstrate a significant difference in peak velocity measurements between right- and left-oriented saccades. This result is also inconsistent with the results obtained by [9], which also demonstrated a significant difference in peak velocity between rightward and leftward saccades. The different results might be related to the fact that the current study did not separate right- and left-eye-dominant participants.

It has been previously demonstrated that prosaccades that are directed horizontally or upwards have shorter latencies compared to saccades directed downwards [11]. In the current study, a significant effect of stimulus spatial position on the latency of saccadic eye movements was observed at all background conditions. The present work observed a similar trend to the results by other studies where saccadic eye movement latencies were higher for downward-oriented saccades comparing to leftward, rightward, or upward saccades [11]. The effects observed in the current study coincide with other studies demonstrating that the latency for vertical saccades is higher than for horizontally directed saccades [14]. As stated by Irving and Lillakas (2019) the direction of the human gaze is more often directed to the left or right and the processing of vertically placed objects differs from the processing of horizontally placed objects. The neural circuit that ensures the analysis of the relations between horizontally placed objects works faster and more efficiently [14]. Other studies examining how the facial stimuli influence the trajectory of saccadic eye movements have observed more pronounced facial effects with downward eye saccades than with upward movements, and also for saccades with longer latencies than for saccades with shorter latencies. This may be due to the relationship between the lower part of the visual field and the space near the face, which makes this area more sensitive to socially significant stimuli [30].

Based on all of the results obtained in the current study, it can be concluded that the best saccadic eye movement performance (lowest latency, highest peak velocity and accuracy) can be observed when the saccades are oriented to the right and to the left. If future studies intend to demonstrate saccadic eye movement stimuli in the vertical direction, it is necessary to take into account that the performance may not be comparable to the average results obtained in the horizontally directed saccades. Future studies should also take into account that contrast dependence of the latency and accuracy of saccadic eye movements is not observed on a medium grey background (both positive and negative polarity) and on a black background (when the contrast levels are not close to the threshold values of subjective perception). Consequently, when presenting a stimulus on a medium grey or black background, stimuli of varying contrasts can be used (if the contrast levels are not extremely low). If the stimuli are to be presented on a light grey background, a very low contrast stimuli should be considered carefully, since the accuracy and latency measurements can worsen in low-contrast conditions.

## Data Availability

The data presented in this study are available on request from the corresponding author. The data are not publicly available due to privacy reasons.
